# MicroRNA-16 Is Down-Regulated in Mutated FLT3 Expressing Murine Myeloid FDC-P1 Cells and Interacts with Pim-1

**DOI:** 10.1371/journal.pone.0044546

**Published:** 2012-09-06

**Authors:** Kyu-Tae Kim, Adam P. Carroll, Baratali Mashkani, Murray J. Cairns, Donald Small, Rodney J. Scott

**Affiliations:** 1 School of Biomedical Sciences and Pharmacy, University of Newcastle, Callaghan, New South Wales, Australia; 2 Department of Oncology and Department of Pediatrics, School of Medicine, Johns Hopkins University, Baltimore, Maryland, United States of America; University of Frankfurt - University Hospital Frankfurt, Germany

## Abstract

Activating mutations in the receptor tyrosine kinase FLT3 are one of the most frequent somatic mutations in acute myeloid leukemia (AML). Internal tandem duplications of the juxtamembrane region of FLT3 (FLT3/ITD) constitutively activate survival and proliferation pathways, and are associated with a poor prognosis in AML. We suspected that alteration of small non-coding microRNA (miRNA) expression in these leukemia cells is involved in the transformation process and used miRNA microarrays to determine the miRNA signature from total RNA harvested from FLT3/ITD expressing FDC-P1 cells (FD-FLT3/ITD). This revealed that a limited set of miRNAs appeared to be affected by expression of FLT3/ITD compared to the control group consisting of FDC-P1 parental cells transfected with an empty vector (FD-EV). Among differentially expressed miRNAs, we selected miR-16, miR-21 and miR-223 to validate the microarray data by quantitative real-time RT-PCR showing a high degree of correlation. We further analyzed miR-16 expression with FLT3 inhibitors in FLT3/ITD expressing cells. MiR-16 was found to be one of most significantly down-regulated miRNAs in FLT3/ITD expressing cells and was up-regulated upon FLT3 inhibition. The data suggests that miR-16 is acting as a tumour suppressor gene in FLT3/ITD-mediated leukemic transformation. Whilst miR-16 has been reported to target multiple mRNAs, computer models from public bioinformatic resources predicted a potential regulatory mechanism between miR-16 and Pim-1 mRNA. In support of this interaction, miR-16 was shown to suppress Pim-1 reporter gene expression. Further, our data demonstrated that over-expression of miR-16 mimics suppressed Pim-1 expression in FD-FLT3/ITD cells suggesting that increased miR-16 expression contributes to depletion of Pim-1 after FLT3 inhibition and that miR-16 repression may be associated with up-regulated Pim-1 in FLT3/ITD expressing cells.

## Introduction

Fms-like tyrosine kinase 3 (FLT3) is expressed and activated in many human leukemias, including a significant percentage of acute myeloid leukemia (AML), and infant/childhood acute lymphoblastic leukemia (ALL) [1], [2], [3]. Activating mutations of FLT3 are found in approximately one third of AML cases and portend a poor prognosis [4]. Internal tandem duplication (ITD) mutations of the juxtamembrane domain coding sequence of the FLT3 gene have been identified in 17% to 34% of patients with AML and 5% of patients with myelodysplastic syndrome [5], [6], [7]. Mutations in FLT3 induce ligand-independent, constitutive activation of FLT3 and activate multiple signaling pathways including up-regulation of Pim-1 [8], [9]. While there is some suggestion that up-regulated Pim-1 may be a consequence of activation of STAT5 in FLT3/ITD expressing cells [8], [10], [11], [12], we hypothesised the presence of a regulatory mechanism involving a FLT3-associated alteration of Pim-1 sensitive miRNA expression.

MiRNA are a highly-conserved family of small non-protein-coding RNA molecules, approximately 22 nucleotides in length, which can negatively regulate their target gene expression post-transcriptionally [13], [14]. This occurs through partial base-pairing at miRNA recognition elements (MREs) within the 3′-untranslated region (UTR) of target mRNAs, resulting in mRNA destabilization and translational inhibition [15], [16]. In recent years the dysregulation of miRNAs has been linked to cancer initiation and progression, indicating that miRNAs may play roles as tumour suppressor genes or oncogenes [14], [17], [18], [19]. Indeed, miRNA profiles can be used to classify human cancers and are surprisingly informative [18], [20], and while the role of miRNAs in apoptosis is not fully understood, evidence is mounting to indicate an important role for miRNAs in this process [21]. In healthy cells, miRNAs are expressed in specific haematological cell types and play important regulatory roles in early haematopoietic differentiation, erythropoiesis, granulocytosis, megakaryocytosis and lymphoid development [13], [22].

Despite the growing evidence for their importance in normal physiology, the regulation of miRNA expression in leukemia is not fully understood [20], [22]. There is an emerging body of research to suggest that miRNAs play an important role in the pathology of haematological malignancies [23], first suggested with the deletion or down-regulation of miR-15 and miR-16 in a large proportion of chronic lymphocytic leukemia (CLL) cases [24]. Subsequent expression profiling studies identified miRNA signatures characterizing CLL outcome [25], [26], ALL [27] and AML associated with various abnormalities [28], [29]. Imatinib treatment of CML patients has also been shown to rapidly normalise the characteristic miRNA expression profile, supporting the notion that miRNAs may serve as a clinically useful biomarker in leukemia patients [30]. Indeed, deletion or down-regulation of miR-15 and miR-16 in CLL is inversely correlated to *BCL2* expression, and both miRNAs have been shown to negatively regulate *BCL2* at a posttranscriptional level [17]. However, only a handful of the potentially hundreds of miR-16 target genes have been identified to date, including CCND1, WNT3A, CAPRIN1, HMGA1, BMI1, WIP1, and SERT (serotonin transporter), though this does suggest an important role for miR-16 in regulating biological processes such as cell cycle regulation, apoptosis, and proliferation [31], [32], [33], [34], [35].

To better understand miRNA regulatory mechanisms in mutated FLT3 expressing cells, we performed miRNA microarray experiments to observe differential expression of miRNAs in FLT3/ITD expressing murine myeloid FDC-P1 cells compared to the control. Our results indicated that a limited set of miRNAs are differentially expressed in FLT3/ITD expressing FDC-P1 cells (FD-FLT3/ITD) when compared to empty vector expressing FDC-P1 cells (FD-EV). We hypothesised that differential expression of these miRNAs may be associated with FLT3/ITD mediated leukemic transformation. Among these miRNAs, we selected miR-16, miR-21, and miR-223 to validate the microarray data by quantitative real-time RT-PCR (QPCR), showing a high degree of correlation. In this study, miR-16 was found to be one of most significantly down-regulated genes in FD-FLT3/ITD cells, and was up-regulated upon FLT3 inhibition. This suggested that miR-16 is acting like a tumour suppressor gene in FLT3/ITD-mediated leukemic transformation. The Pim-1 oncogene has previously been shown to be up-regulated by constitutively activated FLT3, and to play a role in FLT3-mediated cell survival [8], [12], [36], [37], [38], [39], [40]. Further, Pim-1 expression is reported to be down-regulated quickly after treatment of FLT3 inhibitor [8], [12], [36], [37], [38], [39], [40]. It has been suggested that down-regulation of Pim-1 after FLT3 inhibition may result primarily from deactivation of STAT5 in FLT3/ITD signalling and a relatively short half-life of Pim-1 [8], [10], [11], [12]. However, the mechanism of regulation of Pim-1 expression by FLT3/ITD signalling is not clearly understood. Interestingly, we found that computer models from public bioinformatic resources predicted a potential regulatory mechanism between miR-16 and Pim-1 mRNA. Thus, we performed Pim-1 reporter gene assays to determine whether miR-16 may actually interact with Pim-1 *in vitro*. Our results identified that miR-16 appears to bind to the 3′ UTR of Pim-1 and mediate negative regulation of Pim-1 expression. Using quantitative real-time RT-PCR and immunoblotting, we confirmed that reduced Pim-1 mRNA and protein levels were the outcome of miR-16 mimic transfection demonstrating that binding of miR-16 on the 3′ UTR of Pim-1 results in the negative regulation of Pim-1 at a posttranscriptional level.

## Materials and Methods

### Cells, DNA Transfection and Reagents

FLT3/ITD positive human leukemia cell lines, MV4-11 and MOLM-14 cells, were purchased from ATCC (American Type Culture Collection, Manassas, VA). The cells were cultured with RPMI-1640 media supplemented with 10% fetal bovine serum (FBS) at 37°C and 5% CO_2_. The growth factor-dependent murine myeloid cell line FDC-P1 was purchased from ATCC and the cells were cultured with DMEM media supplemented with 10% FBS and murine GM-CSF as previously described [41].

FLT3/ITD expressing FDC-P1 cells (FD-FLT3/ITD) and empty vector expressing FDC-P1 cells (FD-EV) were generated as briefly described below. The construct for FLT3/ITD was originally supplied by Dr. Hitoshi Kiyoi (Nagoya University School of Medicine, Nagoya, Japan). The FLT3/ITD fragment was subcloned into MSCV-IRES-GFP vector and introduced into FDC-P1 cells by retrovirus-mediated gene transfer as previously described [42]. As a control, FD-EV cells were transfected by retroviral particles made using empty MSCV-IRES-GFP vector. FDC-P1 cells expressing either only GFP from empty vector or both FLT3/ITD and GFP were sorted using a FACSAria II cell sorter in order to prepare a homogeneous cell populations. Cell proliferation of FD-FLT3/ITD cells in the growth factor-free media was confirmed by resazurin assay as previously described [43]. Expression of human FLT3/ITD was examined by immune-fluorescence staining using mouse monoclonal antibody against FLT3 (Santa Cruz Biotech, CA) as the primary antibody, sheep anti-mouse IgG conjugated with PE (Chemicon, Billerica, MA) as the secondary antibody, followed by flow cytometric analysis using FACSCalibur Flow Cytometer (BD Biosciences, San Jose, CA). CellQuest software (BD Biosciences) was used for data analysis.

Lestaurtinib (known as CEP-701) was purchased from LC Laboratories (Woburn, MA) and sunitinib was purchased from Chemietek (Indianapolis, IN). The 10 mM stock solutions of each drug were prepared with dimethyl sulfoxide (Ajax Finechem, NSW, Australia) and stored at −80°C. They were diluted into cell culture media immediately prior to use.

### MicroRNA Microarray Assay

Total RNA, including miRNA, for miRNA microarray experiments were extracted by Trizol reagent (Invitrogen, Carlsbad, CA) and purified by miReasy mini kit (Qiagen, Valencia, CA) according to the manufacturer’s protocol. Customized arrays from LC Sciences (Houston, TX USA) were used to obtain differentially expressed miRNAs. Modification of total RNA and consecutive hybridization were carried out on a µParaflo™ microfluidic chip (LC Sciences, Houston, TX USA) according to in-house protocol of LC Sciences [44]. Briefly, the assay started from a 2 µg total RNA sample, which was size-fractionated using a YM-100 Microcon centrifugal filter (Millipore, Billerica, MA, USA), with the isolated small RNAs (<300 nt) 3′-extended with a poly(A) tail using poly(A) polymerase. An oligonucleotide tag was then ligated to the poly(A) tail for subsequent fluorescent dye staining; two different tags were used for the two RNA samples in dual-sample experiments. On the microfluidic chip, each detection probe consisted of a chemically modified nucleotide-coding segment complementary to target miRNA (from miRBase, Version 10.0, http://microrna.sanger.ac.uk/sequences/) and a spacer segment of polyethylene glycol to extend the coding segment away from the substrate. The detection probes were made by *in situ* synthesis using photogenerated reagent (PGR) chemistry. The hybridization melting temperatures were balanced by chemical modifications of the detection probes. Hybridization used 100 µL 6×SSPE buffer (0.90 M NaCl, 60 mM Na2HPO4, 6 mM EDTA, pH 6.8) containing 25% formamide at 34°C. After hybridization detection by fluorescence labelling using tag-specific Cy3 (FD-EV cells) and Cy5 dyes (FD-FLT3/ITD cells), were identified using a laser scanner (GenePix 4000B; Molecular Devices, Union City, CA, USA) and digitized using Array-Pro image analysis software (Media Cybernetics, Bethesda, MD, USA).

### Statistical and Bioinformatic Analysis of Microarray Data

Data were analysed first by subtracting the background and then normalising the signals using a locally-weighted regression (LOWESS) filter [45]. For two colour experiments, the ratio of the two sets of detected signals (log_2_ transformed, balanced) and p-values of the t-test were calculated; differentially detected signals were those with less than 0.05 p-values. To identify miRNA whose expression was significantly different between non-tumour and tumour samples and could identify the different nature of FDC-P1 cells, we made use of ANOVA and class prediction statistical tools. Microarray data were hierarchically clustered using the GeneCluster program (Department of Genetics, Stanford University School of Medicine, Stanford, CA, USA) [46]. Predicted targets for selected miRNAs were analysed using miRanda (http://microrna.sanger.ac.uk/sequences), TargetScan (http://www.targetscan.org) and PicTar (http://pictar.bio.nyu.edu).

### Gene Expression Assay by Real-time PCR

Total RNA, including miRNAs, for quantitative real-time reverse transcription (RT)–PCR (QPCR) experiments were extracted by Trizol reagent (Invitrogen, Carlsbad, CA) and purified by miReasy mini kit (Qiagen, Valencia, CA) according to the manufacturer’s protocol. Using TaqMan miRNA assays kit (Applied Biosystems, CA), reverse transcription was performed and then QPCR was performed specific for each miRNA in triplicate for each sample. TaqMan miRNA assays were performed using an ABI 7500 Real Time PCR System and threshold cycle (Ct) results were calculated using the manufacturer’s software (Applied Biosystems, Carlsbad, CA). Single time-point results were reported as a ΔCt (gene Ct – U6 Ct) after normalization using endogenous control U6, and single time-point gene expression data were reported as 2(−ΔCt) after statistical analysis. The relative expression level was calculated using the comparative Ct method. To quantify Pim-1 and Bcl-2 mRNAs, cDNA was produced from total RNA by treatment with reverse transcriptase using standard procedures. Power SYBR Green PCR master mix from Applied Biosystems (Carlsbad, CA) was used and the data normalized using endogenous control β-actin. Single time-point results were reported as a ΔCt (gene Ct – β-actin Ct), and fold differences were calculated as 2(−ΔCt). Statistical significance between the transfected and non-transfected cell lines was determined using the student’s t-test.

### Reporter Gene Expression

Validation of predicted target genes was accomplished by co-transfecting HEK-293 cells with synthetic miRNA or an LNA-modified antisense inhibitor and recombinant firefly luciferase reporter gene constructs containing 3′ UTR sequences substituted from the target gene as described previously [47], [48]. The pMIR-REPORT™ Luciferase miRNA Expression Reporter Vector (Ambion) contains firefly luciferase under the control of a mammalian promoter/terminator system, with a miRNA target cloning region downstream of the luciferase translation sequence. To test whether miR-16 targets Pim-1, two predicted MREs from Pim-1 were cloned into the pMIR-REPORT multiple cloning site to generate reporter constructs. Lipofectamine 2000 reagent (Invitrogen) was used to transiently co-transfect HEK-293 cells with 4 ng of reporter construct (Pim1_16_1 or Pim1_16_2) and 20 ng of pRL-TK renilla luciferase construct; with 30 nM synthetic miRNA (miR-16 mimic or miR-16 scramble control) or 100 nM anti-miR LNA-modified oligonucleotide (anti-miR-16 or anti-miR scramble control) (Table 1). The Dual-Luciferase Reporter Assay System (Promega) was then used to measure luciferase activity on a BioTek Synergy 2 plate reader. The responsiveness of each reporter-MRE to co-transfected miR-16 or anti-miR-16 was determined by their relative firefly luciferase activities with respect to Renilla luciferase activity (transfection control), normalised against the relative activity with their respective scrambled controls.

**Table 1:** miRNA and anti-miR inhibitor sequences.

Type	Name	Sequence
miRNA^a^	miR-16^+^	UAGCAGCACGUAAAUAUUGGCG
	miR-16^−^	CCAAUAUUUACGUGCUGUUAUU
	miR-16 scramble^+^	AUCCACCACGUAAAUAUUGGCG
	miR-16 scramble^-^	CCAAUAUUUACGUGGUGGAUCG
Anti-miR^b^	Anti-miR-16	CˆGCCˆAATˆATTˆTACˆGTGˆCTGˆCTA
	Anti-miR-16 scramble	CˆGCCˆAATˆATTˆTACˆGTGˆGTGˆGAT

a Synthetic miRNA were designed to mimic the endogenous miRNA, with ‘+’ indicating the mature miRNA strand, and ‘–’ indicating the passenger strand.

b nti-miR inhibitor oligonucleotides were designed complementary to the mature miRNA, with LNA-modified nucleotides preceded by a ‘∧’ symbol.

#### Transfection of miRNA oligonucleotide mimic and Immunoblotting

Target cells were transfected using synthetic miRNAs (Table 1) with Lipofectamine RNAiMAX reagent (Invitrogen) according to manufacturer’s instructions. Following transfection, miR-16 was quantified using TaqMan miRNA assay kit (Applied Biosystems, CA) to confirm over-expression or reduction of miR-16 expression in target cells. QPCR was performed from total RNA extracted from each transfected cell. Aliquots of the transfected cells were washed and lysed with 1% NP-40 lysis buffer to obtain total protein extract. The extracts were loaded onto 10% NuPAGE gels and separated using a Novex Mini-Cell system (Invitrogen, Mt. Waverley, Australia). After electrophoresis and transfer to Immobilon membranes (Millipore, Bedford, MA), Western blotting was performed using antibodies against Pim-1 (12H8, sc-13513, Santa Cruz biotechnology, Santa Cruz, CA) and β-actin (Abcam, Ab8227, Cambridge, MA). Protein bands were visualized using chemiluminescence (ECL Western blotting reagents; GE Biosciences, Buckinghamshire, UK) and imaged on a FujiFilm LAS-3000 Luminescent Image Analyser (Fuji Photo Film, Tokyo, Japan).

## Results

### FLT3/ITD Transformed Growth Factor Dependent FDC-P1 Cells into Growth Factor Independent Cells

**Figure 1:**
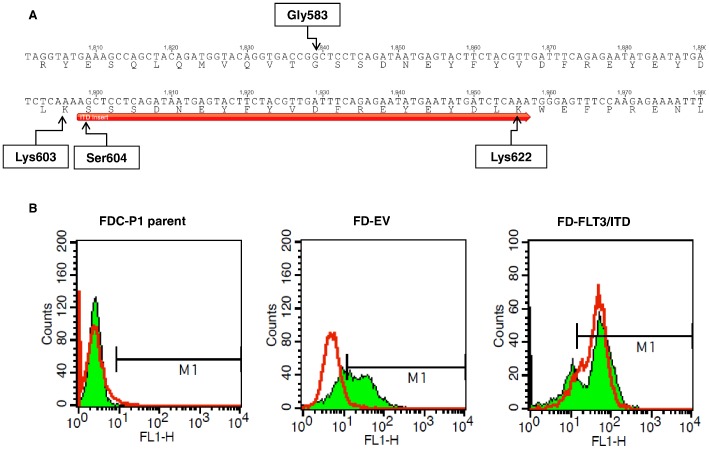
Validation of FLT3/ITD expression in FD-FLT3/ITD cells. (A) Duplication of a 60 bp fragment in the juxtamembrane (JM) domain which resulted in insertion of 20 residues in the JM domain of translated FLT3/ITD. The inserted 20 residues were indicated with a red arrow. Geneious software was used for sequence analysis. (B) Fluorescence from FLT3 (red line) overlaid on GFP (green area) in FDC-P1 parent cells (FD-parent) and FDC-P1 cells transduced either by MSCV-IRES-GFP empty vector (FD-EV) or MSCV-FLT3/ITD-IRES-GFP vector (FD-FLT3/ITD). Expression of human FLT3/ITD was examined by immune-fluorescence staining using mouse monoclonal antibody against FLT3 (Santa Cruz Biotech) as primary and sheep anti-mouse IgG conjugated with PE (Chemicon, Billerica, MA) as second antibody followed by flow cytometric analysis by FACSCalibur Flow Cytometer. Fluorescence data from GFP and FLT3 expression was further analyzed and overlaid on each other using CellQuest software (BD Biosciences).

To understand differential expression of miRNAs associated with FLT3/ITD mediated transformation, we first introduced a human FLT3/ITD expressing vector into murine myeloid FDC-P1 (Factor Dependent Cell Progenitor 1) cells. FDC-P1 cells are murine non-tumourigenic diploid progenitor cells, growth-dependent upon GM-CSF or IL-3 [49], [50]. Full sequencing of FLT3/ITD showed that it contained a 60-bp Internal Tandem Duplication (ITD) mutation in its juxtamembrane domain. It encodes a peptide fragment with the sequence of (SSSDNEYFYVDFREYEYDLK) which is inserted downstream of Lys603 (Fig. 1A). Most of the inserted residues (Ser604 to Lys622) were similar to those in the original sequence of FLT3 (Ser584 to Lys603). However, there was a G to A nucleotide change in the beginning of ITD insert which results in the replacement of Gly583 in the original FLT3 sequence by Ser604 in the ITD insert. This insertion is believed to be rendering the FLT3/ITD kinase domain constitutively active *via* interruption of the autoinhibitory function of the JM domain [51]. We confirmed by flow cytometry that FLT3/ITD was stably expressed in the FDC-P1 cells (FD-FLT3/ITD) after transduction (Fig. 1B). Previously, multiple research groups have reported that constitutive activation of FLT3 tyrosine kinase induced leukemic transformation in growth-factor dependent cell lines [52], [53], [54], [55]. Our cell proliferation assay in growth factor free media also confirmed that the FD-FLT3/ITD cells were factor independent (Fig. S1). Thus, the ectopic expression of FLT3/ITD transformed the cells to growth-factor independence, whereas empty-vector expressing FDC-P1 cells (FD-EV) were still growth-factor dependent for their growth.

### miRNA Expression Profile in FLT3/ITD Expressing Murine Myeloid FDC-P1 Cells Compared to the Control

**Figure 2:**
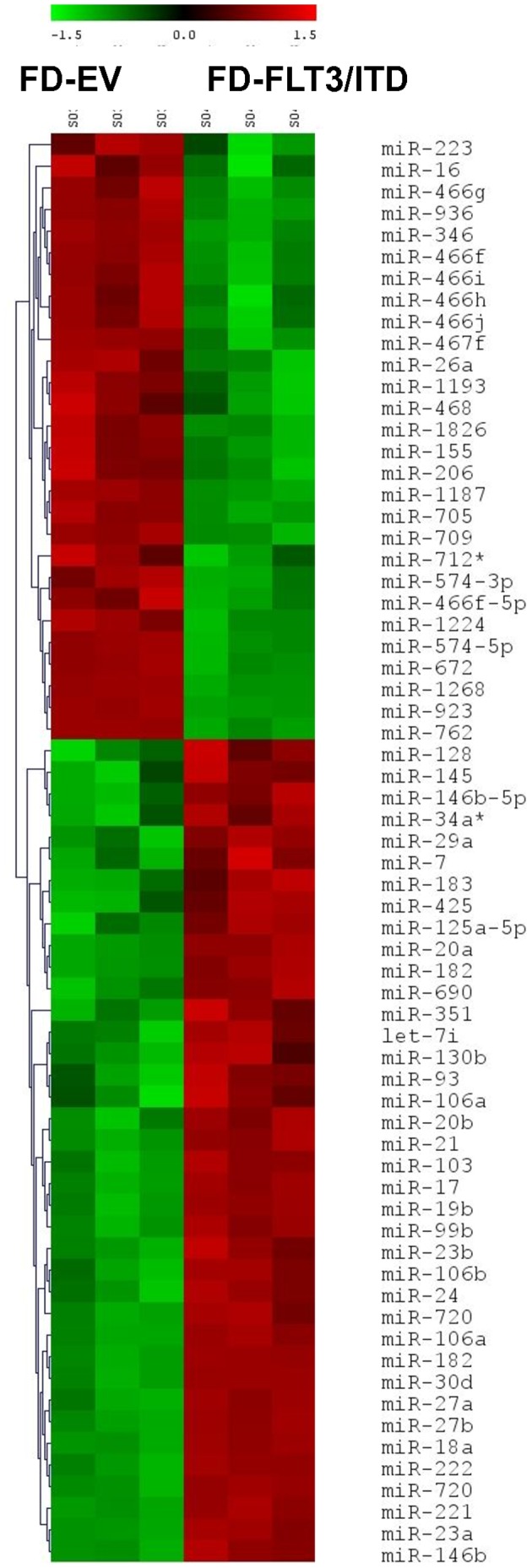
Expression change of miRNAs after tranformation by expression of FLT3/ITD in FDC-P1 cells. Hierarchical clustering of differentially expressed miRNAs in FLT3/ITD expressing FDC-P1 cells (marked as “FD-FLT3/ITD”) comparing control cells transfected by empty vector (marked as “FD-EV”) in FDC-P1cells. Differentially expressed miRNAs were selected (p<0.01) from FD-FLT3/ITD cells compared to the control. A heatmap of expression levels for these miRNAs in the two cell types are presented.

To better understand global miRNA expression signature associated with FLT3/ITD mediated transformation in FDC-P1 cells, we used custom miRNA microarrays to analyse miRNA expression from both FD-FLT3/ITD cells and FD-EV cells (control). RNA from FD-EV cells was labelled with Cy3 and FD-FLT3/ITD cells with Cy5, enabling direct differential expression for each miRNA between the two cell types. A limited set of miRNAs appeared to be affected by FLT3/ITD expression compared to the control group after statistical analysis (p<0.01). (Fig. 2). Several miRNAs including miR-16 and miR-223 were down-regulated after transformation whereas several miRNAs including miR-21 were up-regulated after transformation of FDC-P1 cells by expression of FLT3/ITD. Thus, limited miRNAs were differentially expressed in FDC-P1 cells upon transformation of the cells by the expression of FLT3/ITD.

### QPCR Confirmation of Microarray Data

**Figure 3:**
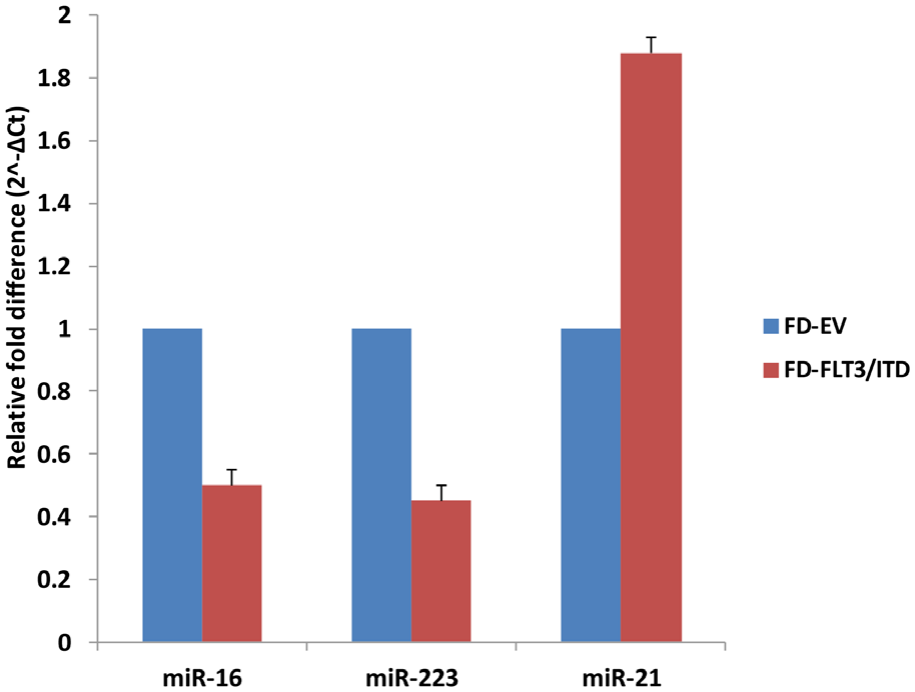
Quantatative RT-PCR validates microarray data. The three miRNAs (miR-16, miR-223, and miR-21) selected from Fig. 2 were analysed by QPCR on total RNA from FD-FLT3/ITD cells. We first obtained ΔCt (gene Ct – U6 Ct) from Ct values of each gene for normalization and then ΔCt values were converted to relative gene expression using the 2^−ΔCt^ method. The graph is presented to show relevant gene expression fold differences in FD-FLT3/ITD cells compared to empty vector expressing control FD-EV cells (fold = 1). The results show the mean and standard deviation for triplicate QPCR results from three independent experiments (p<0.01).

In order to validate the miRNA microarray data, total RNA from FD-FLT3/ITD or FD-EV cells were obtained and then used in quantitative RT-PCR (QPCR) experiments. We selected miR-16, miR-21 and miR-223 on the basis of published reports relevant to the leukaemic phenotype. This demonstrated a high degree of correlation between the microarray data and QPCR data (Fig. 3). We confirmed miR-16 and miR-223 were down-regulated: greater than two-fold lower in FD-FLT3/ITD cells compared to control cells. We also confirmed miR-21 was up-regulated: greater than 1.8-fold higher in FD-FLT3/ITD cells compared to control cells. Thus, the differential expression of our selected miRNAs was validated by QPCR, and correlated strongly with the microarray data.

### mir-16 Increases in Response to FLT3 Inhibition

**Figure 4:**
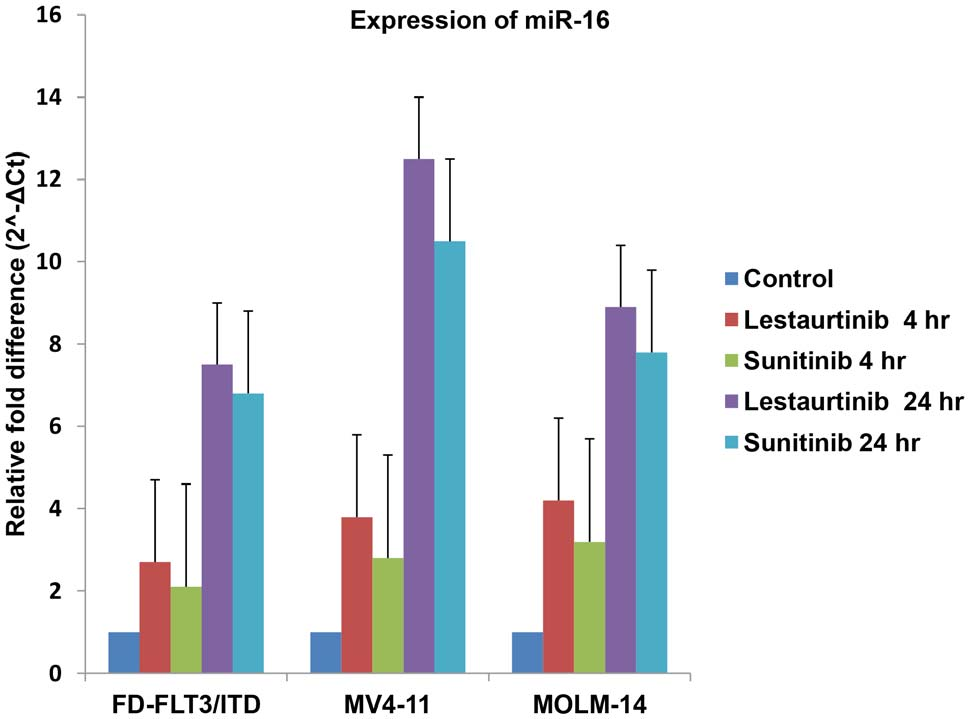
miR-16 expression increases in response to FLT3 inhibition in FD-FLT3/ITD, MV4-11, and MOLM-14 cells. FD-FLT3/ITD cells and FLT3/ITD expressing human leukemic derived cell lines (MV4-11 and MOLM-14 cells) were used. All cell lines were incubated for 4 hr or 24 hr with 50 nM of FLT3 inhibitor (lestaurtinib or sunitinib). RNA was then harvested and used for miR-16 expression analysis by QPCR using TaqMan miRNA assay. U6 small nuclear RNA was used as an internal control to normalize the level of miR-16 expression. Increment of miR-16 expression by 50 nM lestaurtinib or sunitinib with increasing time of treatment compared to control (fold = 1) is presented. The results show the mean and standard deviation for triplicate QPCR results from three independent experiments (p<0.01).

To further validate these findings and investigate the relationship between differentially expressed miRNAs in FD-FLT3/ITD cells and constitutively active FLT3, we performed QPCR before and after treatment of FLT3 inhibitors, lestaurtinib and sunitinib (Fig. 4). Lestaurtinib and sunitinib are reported to selectively deactivate constitutively activated FLT3 [56], [57], [58]. We selected miR-16 for further analysis by QPCR and FLT3 inhibitors as it has been reported to be involved in anti-apoptosis in leukaemic cells. We discovered that miR-16 expression was increased in each FLT3/ITD expressing cell we tested (FD-FLT3/ITD, MV4-11 and MOLM-14 cells) with increasing time points of incubation with FLT3 inhibitors (lestaurtinib or sunitinib) (Fig. 4). Thus, suppressed miR-16 in FLT3/ITD expressing cells appears to be highly associated with activated FLT3 signalling with ITD mutation, and suppression of miR-16 can be reversed by FLT3 inhibition.

### Pim-1 is a Direct Target of miR-16

**Figure 5:**
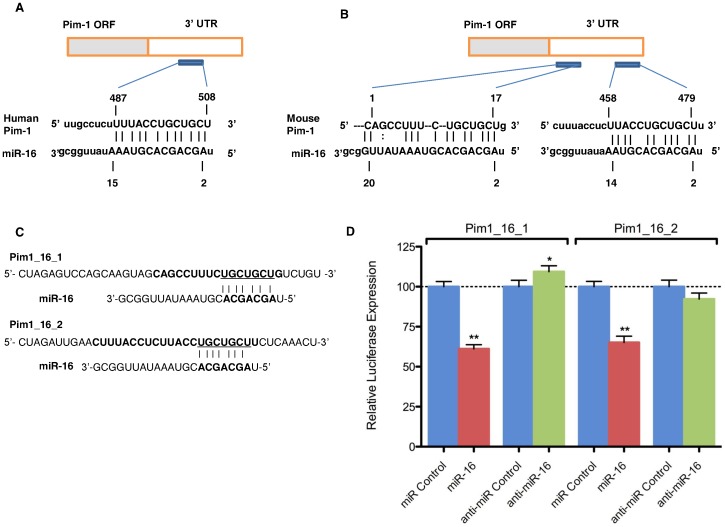
Pim-1 is a direct target of miR-16. (A) Schematic representation of the human Pim-1 transcript. Predicted miR-16 binding site is depicted. The numbers (+487–508) represent the nucleotides (relative to Pim-1 termination codon) that are predicted to base pair with the miR-16 seed sequence. (B) Schemateic representation of the mouse Pim-1 transcript with predicted miR-16 binding site. The numbers are represented like in panel A. (C) Schematic representation of luciferase constructs used for reporter assays. Pim1_16_1 was designed on the basis of +1–17 region of Pim-1 3′UTR and Pim1_16_2 and was designed on the basis of +458–479 region of Pim-1 3′UTR. (D) Luciferase assays in HEK-293 cells transfected with vectors shown in panel C, miR-16 and control oligonucleotides. Bars represent luciferase activity for the corresponding vectors.**, p<0.01; *, p<0.05.

Anti-apoptotic oncogene Pim-1 has been reported to be upregulated in FLT3/ITD expressing cells. In this study, we questioned whether Pim-1 in FLT3/ITD expressing cells may be regulated by one of the miRNAs in the set of miRNAs obtained from our miRNA microarray experiment. Because, like many other mRNAs, Pim-1 mRNA can be targeted by multiple miRNAs, we performed bioinformatic analysis to obtain a list of miRNAs possibly targeting Pim-1 mRNA *via* translational repression. Interestingly, we discovered through bioinformatic computer models that miR-16 may target the 3′ UTR region of both human and mouse Pim-1 (Fig. 5A and B). Indeed, this predicted interaction is conserved in both humans and mice (Fig. 5A and B); with conserved miRNA target sites reported to exert a stronger effect than non-conserved target sits [59], this further strengthened our preliminary hypothesis. Additionally, in the mouse Pim-1 3′ UTR, putative target sites for miR-16 were located in two different locations whilst alignment scores of both sites are equally high (Fig. 5B). To verify Pim-1 as a target of miR-16, we performed luciferase reporter assays with both putative miR-16 binding sites from the Pim-1 3′ UTR (Pim1_16_1 3′ UTR and Pim1_16_2 3′ UTR, Fig. 5C). Our data indicated a miR-16–specific regulation of reporter gene expression of both Pim1_16_1 and Pim1_16_2 constructs (Fig. 5D). Thus, miR-16 possesses two MREs within the Pim-1 3′ UTR that it can bind to regulate Pim-1 expression levels. Taken together, miR-16 appears to bind to the 3′ UTR of Pim-1 and mediate negative regulation of Pim-1 expression.

### Pim-1 is Down-regulated by miR-16 Mimic

**Figure 6:**
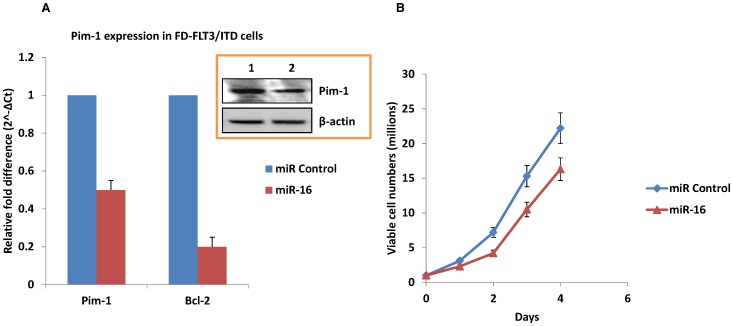
Mir-16 mimic down-regulates Pim-1 and decelerates FD-FLT3/ITD cell growth. (A) Using QPCR, Pim-1 mRNA level was quantified from FD-FLT3/ITD cells upon miR-16 mimic transfection. The graph is presented to show relevant gene expression fold differences in FD-FLT3/ITD cells after miR-16 mimic transfection compared to scrambled miR-16 transfection (fold = 1) as a control. The results show the mean and standard deviation for triplicate QPCR results from two independent experiments (p<0.01). The inset shows the results from immunoblotting of Pim-1 protein upon transfection of miR-16 mimic. Total protein extracts were obtained from FD-FLT3/ITD cells and then subjected to size fractionation in 10% poly-acrylamide gels. Immunoblotting was performed and probed with anti-Pim-1 antibody. Pim-1 protein expression has been reduced in FD-FLT3/ITD cells upon miR-16 mimic transfection (lane 2) when compared to control Pim-1 protein expression (lane 1). The membrane was stripped and re-probed with anti- β-actin antibody to demonstrate equal loading of total protein extract. (B) Overexpression of miR-16 mimic decelerates growth of FD-FLT3/ITD cells. A quantity of 1×10^6^ FD-FV and FD-FLT3/ITD cells were cultured after transfection with synthetic miR-16 mimics for 4 days. Viable cells were counted daily on the basis of trypan blue exclusion. Results shown are the means from triplicate assays. Error bars indicate SD.

The putative target site of miR-16 in 3′-UTR of Pim-1 and luciferase reporter assay results suggested the possibility of inverse correlation of Pim-1 expression by miR-16. To validate increased miR-16 expression can reduce Pim-1 mRNA levels, we analysed expression of Pim-1 by QPCR using total RNA extracted from FD-FLT3/ITD cells upon miR-16 mimic transfection. Indeed, the RNA level of Pim-1 exhibited approximately 2.2-fold decrease upon miR-16 mimic transfection compared to the control (Fig. 6A). Since miR-16 has been shown to target Bcl-2 [17], the RNA level of Bcl-2 was also analysed as a positive control. The RNA level of Bcl-2 paralleled Pim-1 expression upon miR-16 mimic transfection confirming Pim-1 mRNA levels were reduced due to an interaction with miR-16. To determine whether the changes in mRNA result in changes to protein expression, cell lysates of FD-FLT3/ITD cells were immunoblotted by anti-Pim-1 antibody. Our data demonstrated Pim-1 protein levels parallel the observed decreases in Pim-1 mRNA expression. Pim-1 protein also exhibited an approximate 2-fold decrease in FD-FLT3/ITD cells indicating Pim-1 is a direct target of miR-16 and miR-16 induces translational repression of Pim-1 (Fig. 6A, inset). Our data show that enforced expression of miR-16 could not completely deplete Pim-1 expression and reduces both mRNA and protein levels to approximately 50% compared to control cells. This may be due to continuous induction of Pim-1 by the other FLT3/ITD mediated signalling molecules such as STAT5 in FD-FLT3/ITD cells. We next examined whether enforced expression of the miR-16 mimic might result in changes of cell growth. Viable cells were counted daily based on trypan blue exculsion after transfection of synthetic miR-16. It was noted that FD-FLT3/ITD cells transfected with the miR-16 mimic grew more slowly when compared to FD-FLT3/ITD cells transfected with scrambled control (Fig. 6B). Taken together, miR-16 appears to bind to the 3′ UTR of Pim-1 and thus mediate negatively the regulation of Pim-1 expression at a posttranscriptional level, and suppressed miR-16 may contribute to continuous growth of FD-FLT3/ITD cells through up-regulation of anti-apoptotic molecules including Pim-1.

## Discussion

MiRNAs control gene expression by destabilizing targeted transcripts and inhibiting their translation. It is now widely accepted that the expression of miRNAs has been definitively linked to cancer development, and miRNA profiles can be used to classify human cancers. For this reason, a better understanding of differential miRNA expression in malignant phenotypes should provide better development strategies towards more successful therapeutic intervention.

AML accounts for ∼30% of all leukemia diagnoses and has the lowest survival rate. It is a clinically and genetically heterogeneous disease [60], but mutations in the FLT3 gene occur in a large proportion of cases (approximately 30%) and, at least for internal tandem duplication (ITD) mutations, are associated with poor prognosis [7]. FLT3/ITD mutations also occur in 5% of patients with myelodysplastic syndrome (MDS) [5], [6]. Mutations in FLT3 induce constitutive activation of FLT3 and activate multiple signalling pathways and induce leukemic transformation [7], [8], [9]. To better understand the miRNA expression signature in FLT3/ITD mediated cell transformation, we performed retroviral transduction of a FLT3/ITD expressing vector into murine myeloid FDC-P1 cells and used factor-dependent parental FDC-P1 cells transduced with an empty vector (FD-EV) as a control to obtain differential miRNA expressions in FD-FLT3/ITD cells. We confirmed FD-FLT3/ITD cells transformed to growth factor independent cells, as evidenced by continual cell proliferation in the absence of additional growth factors such as GM-CSF. The miRNA signature associated with this transformation was subsequently investigated, revealing the differential expression of several miRNAs in FD-FLT3/ITD cells compared to the control cells (Fig. 2). We selected miR-16, miR-223 and miR-21 from the set of miRNAs for further analysis on the basis of relevancy to the leukemia phenotype and published literatures. To validate microarray data, we performed QPCR using TaqMan miRNA assays on these three miRNA and confirmed that miR-16 and miR-223 were suppressed, whilst miR-21 was up-regulated in FD-FLT3/ITD cells correlating strongly with data from the microarray experiments. In addition to these three miRNAs, we also found several interesting miRNAs, however we were not able to further analyse these miRNAs in this report. For example, one of our suppressed miRNAs from the data in Fig. 2 is miR-155. Previously it has been reported that miR-155 is suppressed in hematopoietic stem cells and plays a role in the blocking of differentiation [61]. In our microarray data, miR-155 was down-regulated in FD-FLT3/ITD cells compared to the control. Thus, suppressed miR-155 may be postulated to be associated with FLT3/ITD mediated blocking of cell differentiation. In contrast to our data, it has been reported that miR-155 is highly up-regulated in FLT3/ITD positive AML patient samples, although the up-regulated miR-155 was independently associated with FLT3/ITD [28], [62]. Thus, further studies of association of dysregulated miR-155 in FLT3/ITD positive AML are warranted to clearly understand role of miR-155 in AML.

It has been reported that aberrant expression of miR-21 is highly associated with various cancer models and appears to be associated with tumour growth [63], [64]. We also found that miR-21 is up-regulated in FD-FLT3/ITD cells suggesting up-regulated miR-21 in FD-FLT3/ITD cells may be involved in anti-apoptosis by targeting molecules for normal apoptosis such as PDCD4 (Programmed cell death 4) [63], [65], [66], [67]. It has been reported that miR-223 regulates normal granulopoiesis [68]. MiR-223 is preferentially expressed in myeloid cells [13], triggers granulocytic differentiation [69], and plays a crucial role in maturation and maintaining granulocytic function [70]. In addition, miR-223 blocks differentiation towards other blood cells such as erythrocytes [71]. It has been reported that CEBPα induces miR-223 expression [72], [73]. Previously, independent research groups reported that CEBPα is suppressed in FLT3/ITD expressing cells [53], [74]. Thus, it can be postulated that suppressing CEBPα may down-regulate miR-223 expression in FD-FLT3/ITD cells. Recently, it has also been found that miR-223 blocks myeloid cell proliferation and is down-regulated in most sub-types of human AML including FLT3/ITD expressing cells *via* targeting the cell-cycle regulator E2F1 [72]. However, we cannot rule out another mechanism of miR-223 mediated leukemic transformation because miR-223 could also be targeting another putative molecule, in addition to E2F1. In our study, we more focused on miR-16 to further analyse its role in FLT3/ITD expressing cells.

Generally, hundreds of mRNAs have been predicted to be potential targets of one miRNA. Numerous target mRNAs of miR-16 have been identified so far [31], [32], [33], [34], [35]. Our results in this report suggest that Pim-1 may be a potential target of miR-16. A few transcriptional regulators for miR-16 such as p53 [75], NF-κB [76] and Myc [77] have been suggested to be involved in regulating the expression of miR-16. Previously, Myc has been reported to be upregulated in mutated FLT3 expressing cells postulating reduced expression of miR-16 is possibly associated with up-regulated Myc [9], [78]. However, further studies are warranted to prove this hypothesis. We discovered that miR-16 is down regulated in FD-FLT3/ITD cells and it is highly upregulated upon FLT3 inhibition in FLT3/ITD expressing FD-FLT3/ITD, MV4-11, and MOLM-14 cells. Thus, our results in this study identified miR-16 is a dysregulated miRNA in FLT3/ITD expressing cells, and suggest that suppressed miR-16 may be associated with regulation of Pim-1 levels in FLT3/ITD signalling. Combined with previous published reports, we hypothesise that Pim-1 may be down-regulated not only by deactivation of its possible transcription factors such as STAT5, but also by increment of miR-16 after treatment of FLT3 inhibitors. As such, it can be postulated that suppressed miR-16 in FLT3/ITD expressing cells may contribute to up-regulation of Pim-1.

To prove our hypothesis of direct interaction of miR-16 and Pim-1, we first performed luciferase reporter assay to determine whether miR-16 can bind to 3′UTR of Pim-1. Our data have shown that miR-16 binds to two 3′-UTR regions of Pim-1 demonstrating miR-16 interacts with Pim-1. We next performed QPCR and immunoblotting to quantify Pim-1 expression upon miR-16 mimic transfection in FD-FLT3/ITD cells. The Pim-1 mRNA and protein were decreased after transfection of the miR-16 mimic demonstrating miR-16 possibly regulates Pim-1 at a posttranscriptional level. We also found that FD-FLT3/ITD cell growth was reduced upon miR-16 mimic transfection showing miR-16 may interact with anti-apoptotic molecules including Pim-1.

Recently, Pim-1 has been reported to be targeted by miR-33a [79]. However, we could not find substantial differences in expression of the miR-33a in FD-FLT3/ITD cells compared to the control. Further, we could not see a substantial change of miR-33a expression upon FLT3 inhibitor treatment in FLT3/ITD expressing cells (MOLM-14 and MV4-11 cells). Thus, miR-33a appears to be not a major miRNA regulating Pim-1 expression in FLT3/ITD expressing cells.

While it is clear that aberrant FLT3 signaling is a major transforming event and plays an important role in leukaemogenesis, it is widely accepted that fully transformed AML has additional “hits” that make FLT3 sufficient to promote disease. Because targeting FLT3 by itself is not sufficient to achieve complete remission or cure in AML patients, it has been suggested that in combination with other modalities it may yield more favourable clinical outcomes [80], [81]. Thus, a combination therapy targeting multiple malignancies could potentially be utilized to override FLT3 inhibitor resistance. Pim-1 has an important role in anti-apoptosis in FLT3/ITD positive cells [8], [36]. Pim-1 may be a putative target for FLT3/ITD for AML to overcome resistance of mono-therapy, as it has been found to be significantly down-regulated on FLT3 inhibition [8]. Furthermore, a recent report [40] suggests that Pim-1 kinase mediates surface expression of CXCR4, which suggests that targeting Pim-1/FLT3 would be a more efficient therapy for targeting the bone marrow microenvironment. For this reason, regulation of Pim-1 level and modulation of miR-16 expression may be important for FLT3/ITD expressing AML.

### Conclusion

In our study, we identified a miRNA signature associated with FLT3/ITD expressing cells. We then confirmed that a selection of these including miR-16, miR-223, and miR-21 are differentially expressed in FLT3/ITD expressing cells. Finally, we focused on miR-16 for further study and found the Pim-1 oncogene, a regulator of FLT3/ITD signalling, is itself potentially regulated by miR-16. Significantly, this provides further evidence that miR-16 expression is an important biomarker of this transformation and provides an alternative target for future drug development.

## Supporting Information

Figure S1 **Cell proliferation assay of FDC-P1 cells transfected by MSCV-IRES-GFP empty vector (FD-EV), or MSCV-FLT3/ITD-IRES-GFP expressing vector (FD-FLT3/ITD) in growth factor-free media.** Equal number of cells was added to 10 wells (in a 96-well plate) containing DMEM media and 10% FCS. The plate was incubated at 37°C with 5% CO_2_ for 48 hours. Then resazurin reagent was added to all wells and incubated for 4 hours. Fluorescent intensity represents the relative number of live cells in each well.
(TIF)
