# Supplementary figures and images for: MicroRNA-16 Is Down-Regulated in Mutated FLT3 Expressing Murine Myeloid FDC-P1 Cells and Interacts with Pim-1
Source: PLoS One. 2012 Sep 6;7(9):e44546. doi: 10.1371/journal.pone.0044546 (PMC3435263; doi:10.1371/journal.pone.0044546)

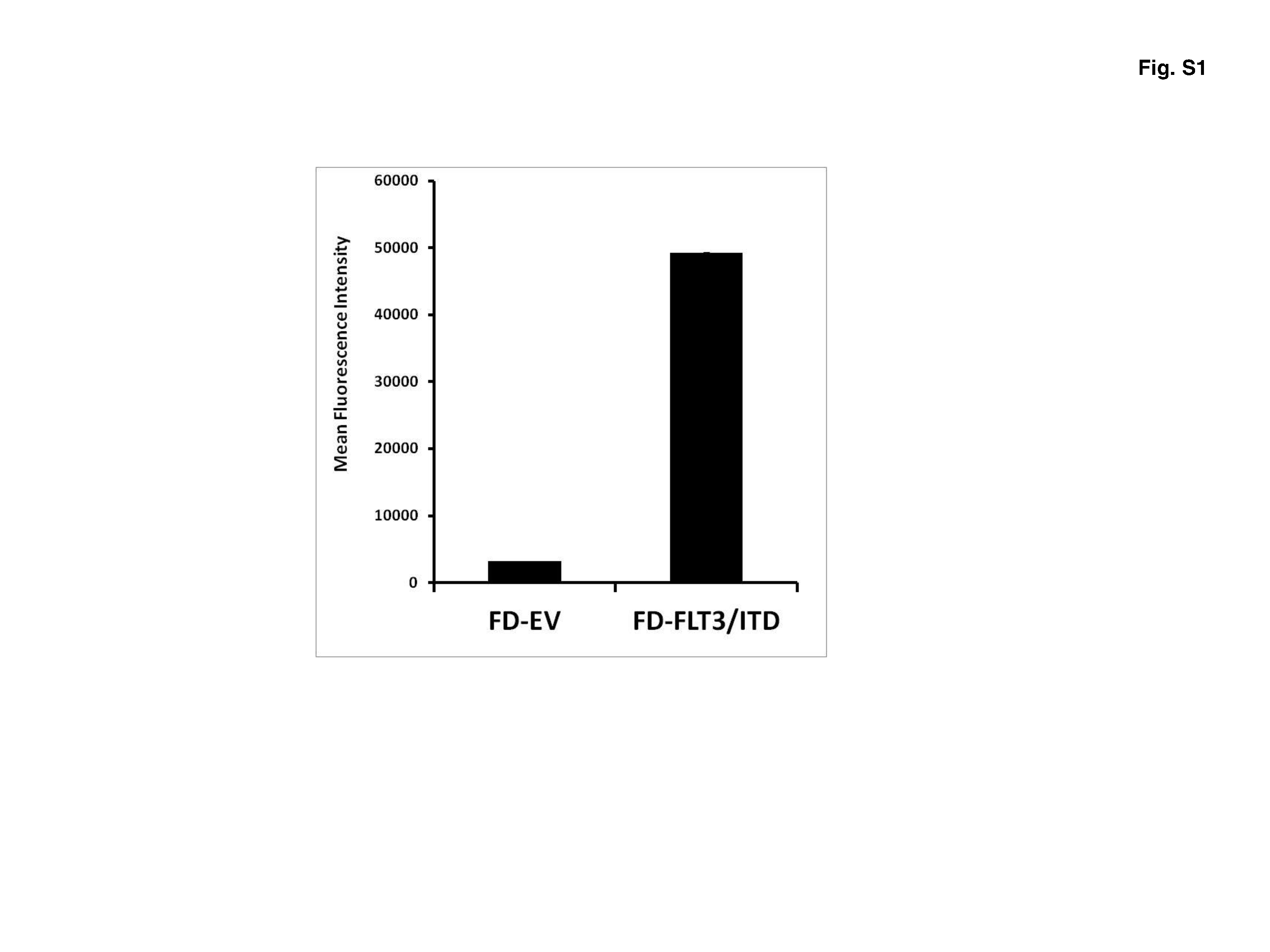

Supplement: Figure S1 — Cell proliferation assay of FDC-P1 cells transfected by MSCV-IRES-GFP empty vector (FD-EV), or MSCV-FLT3/ITD-IRES-GFP expressing vector (FD-FLT3/ITD) in growth factor-free media. Equal number of cells was added to 10 wells (in a 96-well plate) containing DMEM media and 10% FCS. The plate was incubated at 37°C with 5% CO2 for 48 hours. Then resazurin reagent was added to all wells and incubated for 4 hours. Fluorescent intensity represents the relative number of live cells in each well. (TIF) [file pone.0044546.s001.tif]
